# Recent trends in bioinks for 3D printing

**DOI:** 10.1186/s40824-018-0122-1

**Published:** 2018-04-06

**Authors:** Janarthanan Gopinathan, Insup Noh

**Affiliations:** 1 Department of Chemical & Biomolecular Engineering, Seoul National University of Science and Technology (Seoul Tech), Gongneung-ro 232, Nowon-Gu, Seoul, 01811 Republic of Korea; 2 Convergence Institute of Biomedical Engineering & Biomaterials, Seoul National University of Science and Technology (Seoul Tech), Gongneung-ro 232, Nowon-Gu Seoul, 01811 Republic of Korea

**Keywords:** Biomaterials, Bioink, 3D printing, Tissue engineering, Regenerative medicine

## Abstract

**Background:**

The worldwide demand for the organ replacement or tissue regeneration is increasing steadily. The advancements in tissue engineering and regenerative medicine have made it possible to regenerate such damaged organs or tissues into functional organ or tissue with the help of 3D bioprinting. The main component of the 3D bioprinting is the bioink, which is crucial for the development of functional organs or tissue structures. The bioinks used in 3D printing technology require so many properties which are vital and need to be considered during the selection. Combination of different methods and enhancements in properties are required to develop more successful bioinks for the 3D printing of organs or tissue structures.

**Main body:**

This review consists of the recent state-of-art of polymer-based bioinks used in 3D printing for applications in tissue engineering and regenerative medicine. The subsection projects the basic requirements for the selection of successful bioinks for 3D printing and developing 3D tissues or organ structures using combinations of bioinks such as cells, biomedical polymers and biosignals. Different bioink materials and their properties related to the biocompatibility, printability, mechanical properties, which are recently reported for 3D printing are discussed in detail.

**Conclusion:**

Many bioinks formulations have been reported from cell-biomaterials based bioinks to cell-based bioinks such as cell aggregates and tissue spheroids for tissue engineering and regenerative medicine applications. Interestingly, more tunable bioinks, which are biocompatible for live cells, printable and mechanically stable after printing are emerging with the help of functional polymeric biomaterials, their modifications and blending of cells and hydrogels. These approaches show the immense potential of these bioinks to produce more complex tissue/organ structures using 3D bioprinting in the future.

## Background

3D Bioprinting is one of the latest technologies, which is highly used in tissue engineering and regenerative medicine to develop complex tissue structures to mimic native organs and tissues. The bioprinting involves layer by layer deposition of cells-laden biomaterials in a predetermined structural architecture to generate functional tissues or organs. This technique integrates biomaterials, live cells and controlled motor systems for creating complex structures and has shown to have precise control over the developed structures than the other methods which are currently available. Hence, fabrications of very complex structures such as tissue engineering scaffolds with controlled porosity, permeability and mechanical properties, biomedical devices and tissue models are made possible [1–6]. Such complex 3D tissue structures can be designed and developed in computer-aided design (CAD) using the complex geometrical data obtained from the medical imaging techniques such as X-ray imaging, magnetic resonance imaging (MRI) and micro-computerized tomography scan (μ-CT-scan). The advantages of using 3D bioprinting in biomedical field include the development of personalized patient-specific designs, high precision, low cost and on-demand creation of complex structures within a short time [7, 8].

Among the currently employed 3D printing technologies like fused deposition modeling (FDM), direct ink writing (DIW), inkjet bioprinting, selective laser sintering (SLS), stereolithography (SLA) and laser-induced forward transfer (LIFT), the DIW and inkjet bioprinting are frequently preferred for 3D printing of live cells [9, 10]. In DIW, the high viscous solutions or hydrogel or cell suspensions are extruded to obtain 3D structures either with or without a carrier [5]. In case of inkjet bioprinting, low viscous solutions like cell suspensions or colloidal solutions are deposited as droplets at high shear rates (~ 50 μm in diameter) [10–13]. The SLA method is also used in 3D bioprinting, where the curing process takes place without affecting the live cells in the bioink or after printing [14–16]. In addition, LIFT technology is also preferred in 3D bioprinting in a few cases [17–21], where the laser is focused towards a laser absorbing biomaterial layer which helps in developing a local pressure to release ink layer [17, 21]. Also, there are other methods such as acoustic bioprinting, microwave bioprinting, electro-hydrodynamic bioprinting, pneumatic bioprinting, etc. which are currently used for bioprinting of tissues and organs [22]. One of the important components of the 3D bioprinting is the bioink that is used for the printing. This bioink should be highly biocompatible to accommodate live cells, mechanically stable after printing, and it should provide high resolution during printing. Among the different biomaterials, hydrogels are most prominent materials which are used as bioink in the 3D bioprinting. This is mainly due to their ability to hold live cells, modifiable chemical structures, adjustable mechanical and biodegradation properties, and it can yield a good resolution during printing. This review presents the requirements for the selection of bioinks and the properties of the different polymeric biomaterials (natural and synthetic) which are used as bioinks for 3D printing based on their ability to support cell growth, printability, etc. The review covers the different blends and combination of polymeric biomaterials used as bioinks.

### Requirements of bioink for 3D bioprinting

Two important categories of bioink materials are used in 3D bioprinting for developing tissue/organ structures. One is the cell-scaffold based approach and the other one is a scaffold-free cell-based approach [4]. In the first method, the bioink consists of biomaterial and live cells, which are printed to develop 3D tissue structures. Here, the scaffold biomaterial biodegrades, and the encapsulated live cells grow and occupy the space to form predesigned tissue structures. But, in the second method, the living cells are printed directly in a process which resembles the normal embryonic growth. The selected group of live cells forms the neo tissues which are later deposited in a specific arrangement to form fused large functional tissue structures over a time [23]. In case of cell-scaffold based approach, the ideal bioink formulation should satisfy certain biomaterial and biological requirements. Biomaterial properties include printability, mechanical properties, biodegradation, modifiable functional groups on the surface and post printing maturation. Biological requirements mainly include biocompatibility (not only non-toxic to the other tissues/cells, but also live cells’ viability inside bioink), cytocompatibility, and bioactivity of cells after printing. Considering the printability property of the bioinks, it is important to know the processing abilities of the bioink formulation. Also, it should have the ability to self-retain the 3D printed structure after printing. The printability of the bioink depends on the different parameters such as viscosity of the solution, surface tension of the bioink, the ability to crosslink on its own and surface properties of printer nozzle. The printing reliability and the live cell encapsulation highly depend on the hydrophilicity and the viscosity of the bioink solution. If the bioink formulation is highly viscous, then the pressure needed for the extrusion will be more and the flow of the polymer solution from the small nozzle orifice may get affected, e.g.: DIW method. But this higher viscous property possibly will yield 3D structures with higher stability considering the lower viscous solution formulations. The effect of bioink viscosity in 3D printing was studied by Tirella et al. (2009) [24] using a pressure-aided microfabrication method in DIW [24]. They demonstrated the inter-dependence of the viscosity and the printing speed by applying pressure to obtain highly stable printed structures. Moreover, the viscosity of the bioink formulation should be tunable, to facilitate the usage of the same bioink in different commercially available printing machines. In cases of droplet and inkjet based printers, they require a solution viscosity of 10 mPa.s, whereas the extrusion based DIW requires a minimum of 30–6 × 10^7^ mPa.s [9, 10, 22]. However, in laser aided printing, it requires a viscosity of 1–300 mPa.s [7, 22].

Extrusion and droplet based printers which require high viscous formulations as bioink need a characteristic shear thinning property to compensate the high shear stress developed during the printing. The printed structure needs enough stiffness to retain the 3D structure as well as it should support the direct cellular behaviors. As mentioned earlier, the biodegradation of the selected biomaterial should match with that of the tissue of interest, so that once the cells grow and proliferate, eventually they can replace the biodegrading construct with their own regenerated ECMs. Further, the degradation end products and the bioink formulation itself should not create any immunological response in/to the host when implanted in vivo [25]. The bioink materials should facilitate better cell attachment, growth and proliferation inside the 3D construct and it should be convenient to modify the functional groups of the biomaterials to include and deliver different biochemical signals or biomolecules [26]. Apart from these different properties mentioned here, another important property that should be noted, is the stiffness of the print substrate which directly affects the cell survival [25].

Recently, scientists have been exploring the opportunities of using the polymeric biomaterials with more supramolecular functionality as bioinks for 3D bioprinting applications. By using these biomaterials in combination, it may be possible to increase the printing speed, surface properties may be easily tuned to control cell interactions and it can aid in tuning the mechanical properties more precisely by incorporating different gradient biomaterials. The synthesis, characterization and properties of such polymeric biomaterials with supramolecular functionality were elaborated in a detailed review by Pekkanen et al. (2017) [27].

**Fig. 1:**
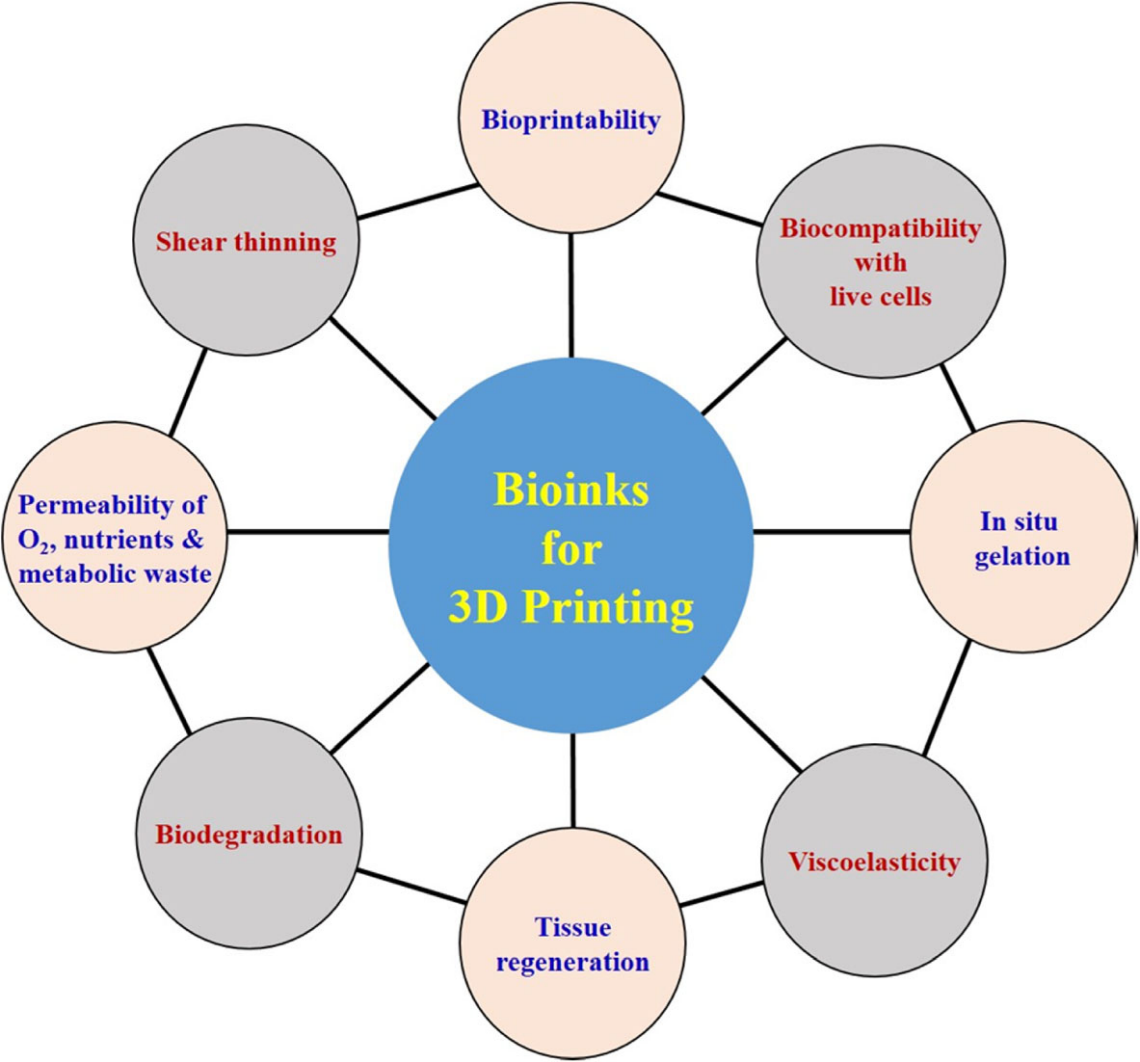
Important requirements for selecting a bioink for 3D printing in biomaterial aspects

Figure 1 shows the important requirements for selecting a bioink for 3D printing in biomaterials aspects. The other important desirable aspects for a bioink include high resolution during printing, in situ gelation, visco-elastic properties, low cost, readily available, industrial scalability, biomimicking the tissue internal structures, mechanical integrity, short post printing time for maturation, and immunological compatibility, when implanted in vivo and wide variety of different types of cells should be employed [23]. Permeability of oxygen gas, metabolic wastes and nutrient transport are also important. These basic requirements are very important while selecting a successful bioink material for 3D bioprinting.

### Bioink biomaterials and their properties

As mentioned in the previous section, the bioink which is used in the 3D bioprinting processes should show important properties and characteristics such as printability and mechanical properties, functionality modifications, controlled biodegradability and non-toxicity to cells (Enable them to get nutrients for their growth and further enhancing their metabolic activity during tissue regeneration) [23]. According to the requirements of the desired tissues and organs, the bioink should be selected and may be modified to regenerate the appropriate tissue structure or organ.

There are many different biomaterials which are reported as bioinks in 3D printing. The following section will discuss about the diverse biomaterials, which are used as bioinks in 3D printing. The polymers which are obtained as a biomaterial from natural resources are called as natural biomaterials in the biomedical fields. These natural materials have varying advantages over synthetic materials, primarily related to the biomimicking of ECM composition or structure, self-assembling ability, biocompatibility and biodegradation properties. Various natural biomaterials which are used as bioinks in 3D printing are discussed in this section. However, synthetic polymers provide their own advantageous properties which are not present in natural polymers such as controllability of mechanical stability, photo crosslinking ability, pH and temperature responses, etc.

### Agarose-based bioinks

Agarose, a marine polysaccharide obtained from seaweed, is one of the highly used biopolymer in the biomedical field for diverse applications because of its excellent gel formation properties [28]. Agarose has a linear polymer chain with an agarobiose repeating unit. This agarobiose backbone chain consists of disaccharides namely D-galactose and 3,6-anhydro-L-galactopyranose [29–31]. Though the gelation, mechanical and biocompatibility properties of the agarose are commendable, however, its ability to support cell growth is limited as reported earlier [32]. Hence, the researchers started using blends of functional biomaterials along with the agarose gel. Kreimendahl et al. (2017) reported the use of agarose-based bioink consisting of collagen and fibrinogen, separately. They demonstrated the ability of these agarose-based blend biomaterials to form stable 3D structures and support endothelial and fibroblast cell growth [33]. In a similar work, where Yang et al. (2017) used agarose/collagen along with sodium alginate as bioink for cartilage tissue engineering application. The bioink was incorporated with chondrocytes and a cartilage like tissue was printed and evaluated in vitro. The printed biomaterial showed enhanced mechanical properties without affecting the gelling behavior considerably [34]. Such high preference to choose agarose in bioprinting by scientists around the world is mainly owing to its excellent gelation properties, biocompatibility and rheological properties which are highly desired in 3D bioprinting [5]. Gu et al. (2016) used agarose along with alginate, carboxymethyl-chitosan to produce 3D printed structures with induced pluripotent stem cells or human-derived neural cells for developing functional neurons. They demonstrated the successful printing and formation of stable 3D structures with cells encapsulated in it [35, 36]. Chemically modified agarose such as carboxylated agarose was used as a bioink to develop mechanically tunable 3D tissue constructs. In their study, the researchers used the hMSCs for its evaluation and the constructs yielded very high cell viability up to 95% than the native agarose gel. The degree of carboxylation can be modified to obtain different gels with varying mechanical properties as per the tissue or organ requirement [37]. Daly et al. compared agarose gel with three other hydrogels as bioink (loaded with mesenchymal stem cells) in 3D printing for cartilage tissue engineering to check their printability and biocompatibility properties towards the differentiation of cartilage or fibrocartilage cells [38]. Among the different hydrogels tested as bioink, alginate and agarose showed higher hyaline like cartilage cell differentiation which showed more type II collagen when stained. Furthermore, all hydrogels showed high hMSCs viability above 80% after printing as reported by the authors [38, 39]. Ozler et al. reported multicellular aggregates used for direct cell 3D printing with smooth muscle, endothelial and fibroblast cells. They demonstrated the ability of these constructs to fuse among one another with high cell viability throughout the study [40]. Even though they have shown good gelation properties, chemical modifications or blending is required for maintaining the 3D printed structure and to enhance more cellular functions.

### Alginate-based bioinks

Alginate is a natural biopolymer attained from brown algae, which is cheap and also called as algin or alginic acid. Alginates are negatively charged polysaccharides, which do not elucidate or provoke much inflammatory response when implanted in vivo. Alginate polymer has two monomers as a repeating unit, namely (1–4)-β-D-mannuronic acid and α-L-guluronic acid. α-L-guluronic acid helps the gel formation, whereas the (1–4)-β-D-mannuronic acid and a combination of (L–4)-β-D-mannuronic acid and α-L-guluronic acid aid in increasing the flexibility of the material [10, 41, 42]. The alginate biopolymers can entrap water and other molecules by using capillary forces and still can allow it to diffuse from inside out. This characteristic property is ideal for 3D bioprinting bioinks [43, 44]. Thus, Zhang et al. (2013) used this alginate-based bioinks with cartilage cells to print hollow constructs. These vessels-like printable microfluidic channels are capable of transporting oxygen, nutrients, biomolecules through the construct and also can support cell growth [45]. In a similar study, Yu et al. (2013) used alginate with cartilage cells as bioinks to develop tubular constructs with a triaxial nozzle assembly. They demonstrated that the ability of the co-axial system with this bioink can support the cartilage progenitor cell viability during printing and post printing processes. This was further confirmed by gene expression studies and other analyses [46]. In another study, Gao et al. (2015) reported a co-axial system which can 3D print high strength constructs with micro-channels for nutrient delivery using an alginate-based hydrogel material [47]. Similarly, Jia et al. (2016) reported an alginate-based blended bioink system which can be directly used to print 3D constructs [48]. Christensen et al. (2015) reported a sodium alginate bioink with mouse fibroblast cells for developing vascular-like structures in a customized 3D printer using calcium chloride crosslinker [49]. Likewise, different polymers were blended with alginate to form various 3D printed constructs for tissue engineering like polycaprolactone (PCL) [38, 50, 51], poloxamer [52] or hydroxyapatite, gelatin [53], etc. Alginates have been used to develop 3D neural tissue constructs. A mixture of different biomaterials such as alginate, agarose and carboxymethyl-chitosan were used to print a 3D construct with stem cells and its in-situ differentiation of stem cells was demonstrated. In a study, Ning et al. (2016) used alginate-based biomaterials for developing 3D constructs with live cells. They investigated the effects of flow behaviors on different cell lines, such as Schwann cells, fibroblast cells and skeletal muscle cells during printing [54]. Also, the temperature and bioink concentration as well as the live cell density were observed to affect the flow rate of the cell suspensions. Alginate was used as the bioink when induced pluripotent stem cells (human) and human embryonic stem cells were bioprinted for the first time. Further, they studied the differentiation of such cells into hepatocyte like cells [55]. Zhao et al. have developed a 3D tissue model of cervical tumor using 3D bioprinting for studying the in vitro biology. For this study, they used both HeLa cells and a combination of bioink consisted of gelatin/alginate/fibrinogen for the printing [56]. Park et al. (2017) reported the effect of different combination of high molecular weight and low molecular weight agaroses and its ability to form 3D structures and support towards live cells. They used fibroblast cells for the in vitro study and demonstrated that a combination of 2:1 ratio high and low molecular weight agarose polymer was good for bioprinting considering their process ability and cell viability studies for soft tissue engineering [57]. Ahlfeld et al. (2017) used synthetic nanosilicate clay for blending with alginate and another polymer named carboxymethyl cellulose (CMC) for developing two different bioink formulations. They tested the bioink samples in extrusion-based 3D plotting technique for creating 3D structures. This approach yielded good printing fidelity and much easier extrusion. The bioink incorporated with immortalized hMSCs printed structures showed above 70% cell viability over 21 days of in vitro culture. The incorporation of such nanosilicate clays further increased the ability of the alginate and CMC samples to release loaded drugs in a more sustained manner. Such addition of nanoparticles in the alginate bioink may enhance the printability and biocompatible properties of the printed structures [58]. In another work, alginate-based nanofibrillated cellulose composite bioinks were compared with hyaluronate-based nanofibrillated cellulose composite bioinks for 3D bioprinting of cartilage tissue construct using induced pluripotent stem cells. Compared to hyaluronate-based bioink, alginate with nanocellulose showed higher cell proliferation and it retained pluripotency for a longer time. They reported that the 3D printed alginates with nanofibrils are suitable for co-culture systems using induced pluripotency stem cells and irradiated chondrocytes [59]. Kosik-Kozioł et al. reported a PLA fiber-reinforced alginate 3D printed cartilage constructs. They showed that the incorporation of PLA fibers increased the mechanical properties (Young’s modulus) of the 3D constructs three folds than the pristine alginate 3D construct. Further, they demonstrated that the fiber-reinforced alginate constructs retained the spherical morphology of the incorporated human chondrocytes cells up to 14 days during the in vitro studies [60]. In another study, alginate was used in combination with different synthetic polymers like 4-arm poly(ethylene glycol)-*tetra*-acrylate (PEGTA) and gelatin methacryloyl (GelMA) for developing biomimetic 3D bioprinted materials for vascular tissue engineering. Initially the bioink was crosslinked using calcium ions ionically (alginate) and then followed by photo-crosslinking (GelMA and PEGTA) to obtain stable structures. PEGTA addition aided the bioink to be modified or adjusted to get the required mechanical or rheological properties for the bioprinting of complex multilayer hollow 3D systems. This combination of bioink provided the favorable environment for the endothelial and stem cells to form highly organized stable perfusable vascular structures. They suggested that this technique may help researchers to obtain better vascularized tissue constructs for tissue engineering applications [48]. These studies clearly show that the alginate based bioink is one of the most preferred materials in 3D bioprinting because of its numerous advantages over the other hydrogels.

### Collagen-based bioinks

Collagen is a main component of ECM, which is obtained from natural biomaterials [61, 62]. Collagen has been used as a bioink material in 3D bioprinting either alone or in combination because of its excellent biocompatible properties [61, 63]. This biopolymer can be crosslinked using temperature or pH change or even by using vitamin Riboflavin [3, 64, 65]. Collagen crosslinking provides them with increased tensile strength and visco-elastic properties than the non-crosslinked collagen [62, 66]. However, the crosslinking or gelation of collagen requires a minimum of 30 mins for gelation at 37 °C. Hence, usage of collagen directly in 3D printing is tough and thus, combining with different other gelation materials may help to address this issue. Further, the mechanical properties of the collagen materials can be increased by adding different polymers in various proportions for using it in 3D bioprinting [66, 67]. Yang et al. (2017) used collagen with sodium alginate as a bioink to develop 3D constructs with chondrocytes. Also, they demonstrated the effectiveness of the combination to suppress the dedifferention of the incorporated chondrocytes to any other cell phenotype and facilitated more cell attachment and proliferation. The results showed improved mechanical properties of the printed construct. Overall, they suggested that a combination of collagen and alginate can be preferred for cartilage tissue engineering applications [34]. In another work, collagen was combined with gelatin (crosslinked separately) to develop 3D constructs using drop-on-demand method. They studied the co-culture of human endothelial cells and hMSCs. They demonstrated the ability of these blended bioinks to produce stable 3D constructs with high biological activity and rheological properties. The inclusion of collagen in the bioink increased the cell spreading and shear thinning of the bioink [68]. Yeo et al. reported a collagen-based, cell-laden bioinks for 3D printing. Their results showed increased mechanical properties and biological enhancement when collagen was used. They adjusted different parameters of 3D printer to obtain such different constructs with varying properties. The post printed samples showed high cell viability than the control alginates. In this study, they used collagen as a core biomaterial and alginate as sheath biomaterial with human stem cells. The new strategy used in this method showed good cell viability and differentiation of hepatocytes from stem cells as desired [69]. Similar work by the same group demonstrated the use of collagen with alginate crosslinked by polyphenol. The bioink containing human adipose stem cells showed higher cell viability and proliferation than the control alginate cell laden bioinks after printing [70]. Further, they reported a similar bioink where they have investigated the effect of preosteoblasts (MC3T3-E1) cells encapsulated tannic acid crosslinked collagen based scaffolds for tissue regeneration [71]. In a recent work reported by Pimentel et al. showed the ability of the transglutaminase-crosslinked gelatin 3D printed constructs for development of vascularized constructs. These constructs may be used for developing complex tumor models and tissue engineering [72]. These different studies related to bioinks for 3D printing show the importance of collagen in this area.

### Hyaluronic acid-based bioinks

Hyaluronic acid (HA) is also a natural ECM which is abundantly seen in cartilages and connective tissues [73]. HA is one of the prominent biomaterials which are used in 3D bioprinting for developing 3D structures. Many different blends of HA-based bioinks are reported till now. One of the work, which explains about the photo-crosslinked HA as bioink to obtain increased rheological properties by using chemical modifications. Like other natural polymers, HA has low mechanical properties and slow gelation behavior, considering the synthetic polymer hydrogels [74]. Ouyang et al. reported a HA-based 3D printed construct using a secondary crosslinking methodology. They demonstrated the capability of the HA-based dual crosslinked bioinks for 3D bioprinting, where it showed no loss in mechanical properties after printing as well as revealed good cellular adhesion properties. The cell adhesion was enhanced by the addition of cell-adhesive oligopeptides in the hydrogels [75]. Recently, Poldervaart et al., showed 3D bioprinting of HA-based hydrogels, which is chemically modified with methacrylate and showed high osteogenic properties. The addition of the methacrylate group enabled them to be cross-linked by photo-crosslinking mechanism. The printed constructs showed enhanced mechanical properties, high stability after printing, but exhibited negligible reduced cell viability when tested with hMSCs [76]. In another study, HA was combined with different synthetic polymers to obtain more stable structures with high cell viability. They demonstrated the ability of the hybrid 3D printed structures to enhance chondrogenesis using a thiol linked HA/polyglycidols gel with PCL. Both chemical and photo-crosslinking were used in this study to enhance the functional properties of the bioinks [77]. A detailed review about the advantages of the HA as biomaterials in various tissue engineering applications, can be observed from a review paper by Hemshekhar et al., 2016 [78]. The different combinations with synthetic polymers and its properties as injectable gels were discussed in detail. Multiple cell types are bioprinted by using a combination of HA and various polymers in 3D bioprinting [23]. Sakai et al. (2017) reported HA-gelatin based bioinks which can be polymerized using visible light with the help of Ruthenium-based complexes. They demonstrated the ability of these biomaterials to enhance cell viability and differentiation of human adipose stem cells [79]. In another recent work, highly tunable HA-carboxymethylcellulose gels were reported. The mechanical properties and cell viability of the different concentrations were analyzed, suggesting that high concentration may yield higher cell viability and stability to the 3D printed structures [80]. These recent studies show the advantages HA as bioinks in 3D bioprinting technology.

### Various other bioinks used in 3D printing

The numerous bioinks used in 3D printing including fibrin, cellulose, silk, ECM-derived bioinks, cell aggregates, cell spheroids, etc. are described below in detail.

#### Fibrin-based bioinks

Fibrin is a protein which is seen in the blood and helps in clotting. Fibrin hydrogel can be made from fibrinogen by enzymatic treatment of thrombin. This hydrogel has excellent biocompatibility and biodegradation properties, but it has weak mechanical properties [81]. Zhang et al. used fibrin hydrogels along with PCL/PLCL to develop 3D constructs of urethra and seeded multiple cell types to investigate the in vitro effects of this material [82]. In another work reported by England et al. (2017), where fibrin was used with HA hydrogels to encapsulate Schwann cells and used to 3D printing. In vitro characterizations and ability of the bioink to support nerve regeneration were investigated [83].

#### Cellulose-based bioinks

Carboxymethyl cellulose (CMC) is a semi flexible polysaccharide obtained from cellulose [84]. CMC can be converted into an environment-sensitive hydrogel by altering its concentrations, molecular weight, salts and degree of methyl grafting appropriately [85]. The aqueous solution of CMC can form gels below 37 °C [86]. For bone regeneration, CMC along with bioactive glass was to develop 3D constructs with high mechanical properties [87]. Markstedt et al. also reported a nanocellulose alginate-based bioinks for cartilage tissue engineering with improved cell viability and mechanical properties of the printed 3D constructs [88]. In another work reported by Ávila et al., the nanocellulose hydrogels were used for developing patient-specific auricular cartilage tissue from 3D bioprinting method. Those constructs showed excellent shape, size retention and high cell viability after printing. In addition, redifferentiation of human nasal chondrocytes to form neo-cartilage specific ECM substances was achieved [89]. Markstedt et al. reported a cellulose nanofibrils and cross-linkable xylans-based inks for 3D printing with high mechanical integrity and excellent printing properties [90]. Nguyen et al. compared the ability of nanocellulose with alginate and HA as bioinks in 3D bioprinting for cartilage tissue engineering. They demonstrated that the nanocellulose with alginate combination showed better cell viability and differentiation of the induced pluripotent stem cells after printing than with HA [59]. In a review by Sultan et al., different nanocellulosic biomaterials and its blends which are used as bioink for 3D printing were elaborated in detail [91].

#### Silk-based bioinks

Silk fibroin is a natural protein obtained from silk worm. These silk-based scaffolds are more frequently used in regenerative medicine and tissue engineering because of its exceptional properties [92]. Das et al., 2015, reported a silk-gelatin based bioink for 3D bioprinting of cells laden constructs. They used mesenchymal progenitor cells in the bioink formulation and cross-linked the silk-gelatin combination using two methods, i.e. sonication and enzymatic crosslinking [93]. In a similar work, Rodriguez et al. demonstrated that the use of silk and gelatin as a bioink for enhancing the biocompatibility, cell permeability and tissue integration in soft tissue reconstruction. In this study, they used glycerol as a physical cross linker [94]. Silk fibroin protein with alginate was used as bioink in Inkjet printing. The alginate was cross-linked using calcium chloride, and the tyrosine residues of silk fibroin was cross-linked using horseradish peroxidase after printing the construct [95]. Xiong et al. demonstrated the efficacy and mechanism of gelatin-silk based ink to regenerate skin. They demonstrated enhancement of the granulation and tissue regeneration in both in vitro and in vivo by incorporating fibroblast growth factor-2 in the ink before printing [96]. Zheng et al.*,* (2018) reported free standing silk-based bioinks consisting of PEG in the composition. These biomaterials showed excellent printability with high resolution and supported MSCs viability for a longer period. Also, they suggested that use of higher silk content increased cell viability to a large extent [97]. Recently, spider silk is also getting more attention because of its excellent mechanical properties. In a related work, DeSimone et al. used recombinant spider silk proteins in developing 3D printing bioinks. The spider silk protein was thermally gelled along with mouse fibroblast cell lines. Even though printed constructs showed less cell viability in spider silk protein based bioinks, when it was added with gelatin, the results were promising. Hence, to further improve and enhance the cell viability properties, addition of biocompatible materials in silk may increase the quality of the printed materials [98].

#### Extracellular matrix (ECM)-based bioinks

ECM is the mixture framework which consists of different components such as collagen, glycosaminoglycans, chondroitin sulphate, elastin, etc. where cells are present. Decellularized ECM (dECM) materials are obtained from the desired tissues where cells are removed by a sequential procedure leaving the ECM intact [99]. The obtained constituents are crushed to form a powder-like state and dissolved in a buffer solution and used as bioink for 3D printing. Further, to enhance the printability of the dECM-based bioink, different polymeric hydrogels may be added to the solution. Pati et al. used PCL to improve the printability of the dECM bioink obtained from different tissue types and used it for 3D printing of tissue constructs by cells. The bioink formulation can be dissolved in an acidic buffer and pH of the solution may be adjusted to prevent cell damage. The investigation showed high cell viability and functionality of the constructs after the printing [100]. Further, the same group developed another method for dual crosslinking of the dECM biomaterials using the vitamin-B2 as a covalent crosslinker and photo crosslinking using UV light. The 3D printed constructs showed high cell viability and cardio-myogenic differentiation [101]. The dECM was used as bioink in 3D bioprinting for developing cell-laden 3D constructs for tissue engineering applications. The researchers developed a 3D system which can precisely control the heating and pH of the bioinks which enable them to form gels at 37 °C while printing. They demonstrated that the precise stacking of such cells by the system did not affect the cell viability, even while mild heating it did not induce any harmful effects to the printed cells [102]. Jang et al., (2017) reported 3D printing of dECM with dual stem cells for cardiac patch development. The constructs were able to form fast vascularization with cell viability for longer time [103]. Even though dECM provides good cell viability and functionality, the isolation and quantification of DNA and ECM constituents from the desired tissue are costly when compared to other hydrogel bioink formulations used for 3D bioprinting.

#### Cell aggregates as bioinks

3D printed constructs were developed using a bioink consisting of spherical cell aggregates (spheroid) with several thousands of cells. The spheroids were dispensed one by one into scaffolds which are biocompatible, and they were allowed to fuse by the self-assembly process. Further, they studied the structure formation by computer simulation studies [104]. Yu et al. demonstrated a novel tissue spheroids bioink for 3D bioprinting constructs without using any scaffolds. They were able to form tissue strands up to 8 cm long with rapid fusion of the cells and mainly, by a self-assembly process without using any harsh chemicals as crosslinker or as support materials. These structures formed native tissue like constructs with a promising application in articular cartilage tissue engineering [105]. In another work, the researchers prepared the cell aggregates or cell sheets using a thermo-sensitive polymer gel as substrate. The poly(N-isopropyl acrylamide) was used as the temporary substrate for the cells. After successful cell growth onto the substrate, the cell sheets were detached by applying mild heat without disturbing the cell-matrix arrangement. The detached cell sheets were separated and used as bioinks for 3D bioprinting constructs. These bioinks showed better results than the normal cell aggregates, because it preserved the ECM intactness. These results are more promising, considering the cell viability and other analyses carried out in this work [106].

#### Synthetic biomaterials as bioinks

Even though natural polymers or hydrogels provides the desired microenvironment mimicking the native ECM for cell attachment and proliferation, the tunable properties of the natural polymers are low [107]. Hence, these natural polymers are combined with either synthetic or another natural polymer to obtain more stable structures with tunable properties for the 3D bioprinting. Even though the synthetic polymers may not promote cellular adhesion or promotion as natural polymers, they are promising candidates to tune the properties to improve the mechanical properties, printability, cross linking, etc. [108]. In synthetic polymers, Pluronic and poly(ethylene glycol) (PEG) are the most commonly used polymers in 3D bioprinting. Pluronic is a block copolymer, consisting of two hydrophobic groups and a hydrophilic group in between them. The advantage of using Pluronic in 3D orienting is mainly due to its ability to form self-assembling gels at room temperature and it can flow at 10 °C [109]. Wu et al. printed microchannel using Pluronic in photo-polymerizable polymer and developed microvascular structures [110]. Similarly, another group of researchers used acrylated Pluronic to develop UV cross-linked 3D constructs which are more stable [111]. Among the PEG-based bioinks, the PEG-diacrylate and methacrylate are the widely used polymers in extrusion-based 3D printing [112–114]. PEG has been used with different materials as blends in 3D printing to increase the mechanical properties of the constructs [115]. Many polymers such as alginate, collagen, etc. were combined to form different bioinks and to tune the properties as per the requirements [116–118]. Mozetic et al. (2017) reported a blend of Pluronic and alginate to investigate its effects on myoblast cell viability and alignment. Further gene expression confirmed the improved viability compared to the normal 2D cultures [119]. There are many other synthetic polymers which are used as bioinks in 3D printing applications. Different types of bioinks and their various properties related to 3D printing are listed in the Table 1.

**Table 1:** Recent works on natural and synthetic polymeric bioinks and their various properties related to 3D printing

Bioink polymers	Bioink concentration	Cell density & viability	Gelation method	Temperature used	Tested cell types	Days	Applications	Advantages (A) & disadvantages (DA)	Reference
Agarose & its blends	20 mg/mL	2.5 × 10^5^ /mL > 70%	Thermal /ionic	30–40 °C	Bone marrow stromal cells (BMSCs)	7	Bone - tissue eng (TE)	A: Better mechanical strength, low-price DA: Inferior cell adhesion	[40, 120–122]
Agarose-based gel	2% (w/v)	70:25:5 ratio of 3 cell types 83–97%	Thermal & ionic x-linking	4 °C to 70 °C	Smooth muscle cells, human endothelial cells, and NIH 3 T3 fibroblasts	10	Vascular TE		
Agarose & its blends	3% (w/v)	1.6 × 10^5^/mL 98.8%	Thermal x-linking	37 °C	MSCs	21	Adipose & bone TE	A: Quick gelation, low price, high shape integrity DA: Inferior cell adhesion, Clogging	[32, 49, 121–123]
Alginate	1%	5 × 10^6^/mL 90.8%	Ionic crosslinking	37 °C 37 °C 37 °C	NIH3T3 fibroblasts	1	Vascular TE		
Alginate	1–2%	2.5 × 10^5^/mL 95%	Ionic x-linking	40 °C	Bone marrow stromal cells	10	Bone TE		
Collagen	0.223%	1 × 10^6^/mL 95%	pH-mediated	37 °C	Dermal fibroblasts	8	Perfusable artificial tissue	A: Enhance cell adhesion factors like RGD DA: Low mechanical stability, high cost, gelation slow, clogging	[34, 121, 122, 124]
Collagen/alginate	15 mg/mL collagen 0.1 g/mL alginate	1 × 10^7^/mL 90%	Ionic	RT	Primary chondrocytes	21	Cartilage TE		
Gelatin	10–20%	5.9 × 10^5^ /mL 91%	Thermal	60 °C	Fibroblasts	8	TE, stem cell, & cancer research	A: Enhances cell attachment, reversible, low cost DA: Low stability, delicate, less functionalities w/o chemical alteration	[72, 121, 122, 125, 126]
Gelatin/ Agarose	0.06%	1 × 10^7^/mL 84.6%	Ionic x-linking	37 °C-RT	NIH3T3	7	Heart valve regeneration		
Gelatin with transglutaminase (TG)	5% (w/v) with 2.5–20 units of TG/g of gelatin	1 × 10^7^/mL High	Thermal & chemical	4–37 °C	HepG2	15	Tumor modeling & regenerative medicine.		
Hyaluronic acid (HA) with gelatin	0.5% (w/v) HA-Ph, 3.0% (*w*/*v*) Gelatin-Ph, Ru(II) & SPS	3.0 × 10^5^ /mL > 95%	Photoinitiated gelation	25 °C	Human adipose stem cells (hADSCs)	25	TE & regenerative medicine	A: Enhances cell growth, angiogenesis, quick gelation, high shape integrity DA: Fast degradation, low mechanical properties	[79, 80, 122, 127]
HA with methylcellulose (MC)	2.0 wt% HA, 5–9 wt% of MC	25:1 ratio > 75%	Thermal	4–37 °C	MSCs	15	TE		
Fibrin	10 mg/ml, 20 U/ml	2 × 10^6^/mL 74.27%	Fibrinogen-thrombin	37 °C	NT2 neurons	15	Nerve TE	A: Enhances angiogenesis, quick gelation DA: Clogging, low mechanical properties	
Silk/PEG	5–10% w/v	2.5 × 10^6^ /mL Good	Thermal, chemical	37 °C	Human bone marrow MSCs & fibroblasts	84	TE	A: Low cost, good cell adhesion DA: Modification required, long process	[97, 122]
Decellularized the adipose (adECM), cartilage (cdECM) & heart (hdECM)	3%	1 to 5 × 10^6^/mL > 90%	Thermal & pH	37 °C	Human adipose-derived stem cells (hASCs) & human mesenchymal stromal cells (hTMSCs)	14	TE, in vitro drug screening & tissue/cancer model.	A: Resemble ECM, high cell adhesion DA: Medium shape integrity, tissue availability, long procedure	[100, 103, 122]
HdECM with vitamin B2 & VEGF	20 mg/Ml	5 × 10^6^/mL 90–95%	Photo- & thermal- polymerization	37 °C	Human c-kit + cardiac progenitor cells (hCPCs), MSCs	56	Cardiac TE		
Pluronic F127	1.6 mmol/g	2 × 10^7^/ml 91.3%	Photo-polymerization	37 °C	Bovine chondrocytes	7	Cartilage TE	A: Reversible polymer, synthetic DA: Low mechanical properties, fast degradation	[49, 111, 121, 122],
Pluronic PF127 /alginate	2 wt% alginate with 20 wt% Pluronic	2 × 10^6^/mL 85%	Ionic	37 °C	Murine C2C12 cells	21	Muscle TE		
PEG & peptides	10% w/v	6 × 10^6^/mL 90%	Photo-polymerization	RT	hMSCs	21	Bone & cartilage TE	A: Good compatibility when blended DA: UV curing, low cell adhesion and mechanical properties	[47, 122]
Gelatin Methacrylate (GelMA)	10–20%	1.5 × 10^6^/mL 97%	Photo-polymerization	27–30 °C	HepG2	14	Liver TE	A: Easy degeneration, mechanically strong, blending possible, printability high DA: Photo-polymerization may affect cell, delayed gelation	[122, 128–131]
Gelatin Methacrylate (GelMA)	10%	1.5 × 10^7^/mL 75% to 90%	Photo-polymerization	37 °C	Articular cartilage- chondroprogenitor cells ACPCs & MSCs	52	Cartilage TE		
GelMA & Gellan gum	3–20% with 0–1.5% gellan gum	10–20 × 10^6^/mL Good	Photo-polymerization	15–37 °C	Equine chondrocytes	42	Cartilage TE		
GelMA, GelSH & heparin	10% with 1% of heparin	15 × 10^6^/mL 74–86%	Photo-polymerization	RT	Chondrocytes	35	Cartilage TE		

## Conclusions and future perspectives

3D bioprinting has the robust capabilities to produce tissue/organ structures with ease; however, it needs further enhancements in different areas such as bioinks, commercialization of the 3D printed products, etc. This method can facilitate to develop more complex patient-specific 3D structures for urgent medical needs. It has numerous advantages like design flexibility, printing modes, use of specific cell lines, control of biodegradation and mechanical properties, etc. Among the different approaches, the cell-laden hydrogels are highly used for developing such 3D structures. The different selection criteria for bioinks and various available bioinks and their properties were discussed in this review. The development of ideal bioink is still in progress and owing to the significant contributions from around the world, it may be possible to use this technology for commercial applications in the future. Even though cell laden biomaterial bioinks are highly used, ECM-based bioinks, decellularized bioinks, cell aggregates or spheroids are also showing promising results towards the development of functional tissues or organs using 3D bioprinting technology. However, these techniques need very large number of specific cells which limits its use in different tissues and organs. Apart from the bioinks, it is also considered that the development of advanced bioprinters with high resolution and less cost may further enhance the prospects of this research area. In case of bioink selection and usage, many novel biomaterials with supramolecular functionality, reversible crosslinking polymers, stimuli-responsive hydrogels are reported recently, which are more promising. The future of bioinks and 3D bioprinting is promising, leading to the development of advanced patient-specific tissue/organs and devices in the future.

## Abbreviations

3D: Three dimensional
CAD: Computer aided design
CMC: Carboxymethyl cellulose
CT: Computed tomography
dECM: Decellularized ECM
DIW: Direct ink writing
DLP: Digital light processing
ECM: Extracellular matrix
FDM: Fused deposition modeling
HA: Hyaluronic acid
hMSC: Human mesenchymal stem cells
LIFT: Laser-induced forward transfer
MRI: Magnetic resonance imaging
PCL: Polycaprolactone
PEG: Poly(ethylene glycol)
PLCL: Poly(lactide-*co*-caprolactone)
SLA: Selective laser sintering
SLA: Stereolithography
UV: Ultraviolet rays
μ-CT-scan: Micro-computerized tomography scan
